# circHIPK3 regulates cell proliferation and migration by sponging microRNA-124 and regulating serine/threonine kinase 3 expression in esophageal squamous cell carcinoma

**DOI:** 10.1080/21655979.2022.2060776

**Published:** 2022-04-21

**Authors:** Da Yao, Shengcheng Lin, Size Chen, Zhe Wang

**Affiliations:** a Department of Thoracic Surgery, Shenzhen Second People’s Hospital. The First Affiliated Hospital of Shenzhen University, Shenzhen, Guangdong, China; b Department of Thoracic Surgery, National Cancer Center/National Clinical Research Center for Cancer/Cancer Hospital & Shenzhen Hospital, Chinese Academy of Medical Sciences and PeKing Union Medical College, Shenzhen, Guangdong, China

**Keywords:** ESCC, circHIPK3, miR-124, AKT3, proliferation, EMT

## Abstract

Circular RNAs (circRNAs) are a type of important non-coding RNAs that widely involve in the physiological and pathophysiological process. Recent research has established a link between circHIPK3 and the malignant activity of cancer cells. However, circHIPK3’ role in esophageal squamous cell carcinoma (ESCC) still needs more focus. To determine the prognostic value of circHIPK3 in patients with ESCC, the expression of circHIPK3 was quantified in 32 pairs of ESCC using real-time polymerase chain reaction (RT-qPCR). Then, the correlation between circHIPK3 expression and clinical characteristics of patients was also analyzed. The function of circHIPK3 in the development of ESCC was investigated using cell biology studies and bioinformatics. The results showed that the expression of circHIPK3 was considerably higher in tumor tissues from ESCC patients than that of adjacent tissues, which was associated with a poor prognosis. Additionally, silencing of circHIPK3 expression retarded esophageal cancer cell proliferation, migration and epithelial-mesenchymal transition (EMT) *in vitro*, as well as the growth *in vivo*. Mechanistically, we discovered that circHIPK3 behaved like a sponge, absorbing microRNA-124 (miR-124) and promoting serine/threonine kinase 3 (*AKT3*) expression. Our findings indicate that circHIPK3 acts as an oncogene in ESCC and that the circHIPK3-*AKT3* axis may be a therapeutic target for patients with ESCC.

## GRAPHICAL ABSTRACT

**Figure:**
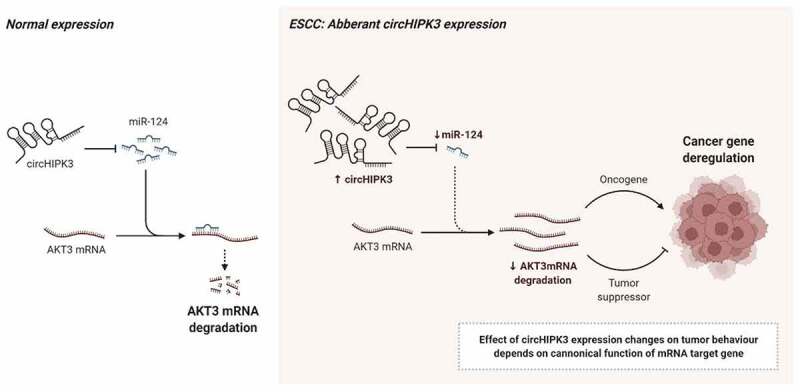

## Highlights

1. The expression of circHIPK3 is upregulated in ESCC
2. Elevated circHIPK3 expression correlates with clinical features of patients with ESCC
3. CircHIPK3 regulates cell proliferation and migration of ESCC
4. CircHIPK3 sponges miR-124 in ESCC
5. CircHIPK3 regulates AKT3 expression vis sponging miR-124

## Introduction

Esophageal cancer is one of the most frequently occurring types of cancer in humans with a significant fatality rate [1], especially with a high incidence and over 150,000 individuals dying each year in China [2]. Esophageal cancer is characterized by a high rate of metastasis and invasiveness and a dearth of early detection markers, which makes it is typically identified in the middle or late stages. As a result, the 5-year survival rate of esophageal cancer is about 15% to 25% [3,4]. Esophageal cancer histologically classified as esophageal squamous cell carcinoma (ESCC) and esophageal adenocarcinoma. The latter is more prevalent in European and American countries, whereas ESCC accounts for around 90% of esophageal cancer cases in China [3,5]. Therefore, elucidating the molecular mechanisms behind the occurrence and progression of ESCC and enhancing early detection can anticipate to increase the cure rate and decrease the death rate associated with esophageal cancer.

Circular RNAs (circRNAs) with the closed-loop structure own highly stable and conservative biological genetic features that are involved in a wide variety of physiologic and pathological processes [6]. It has been identified that circRNAs not only regulate cell apoptosis and metabolism, but also function as oncogenes or tumor suppressor genes [7]. Notably, the most common biological function of circRNAs is as a ‘molecular sponge’ for miRNA to impair the cell’s basic activity [6]. For instance, circSLC7A6 can serve as a ceRNA of miR-107 to upregulate ABL2 expression that promotes the progression of Wilms’ tumor [8]. Circ_0005320 facilitates oral squamous cell carcinoma tumorigenesis via sponging miRNA-486-3p and miRNA-637 [9]. Circ_0000654 that absorbs miRNA-149-5p to upregulate inhibin-beta A enhances the growth of gastric cancer cells [10]. Moreover, Zheng Q et al [11] has discovered that circHIPK3 is the most abundant among the three distinct types of splicing bodies in the circRNAs of the HIPK3 gene, which plays a critical role in cells growth through sponging multiple miRNAs. Numerous investigations have revealed substantial differences in the expression of circHIPK3 between human tumor cells and normal cells [12–14], which indicate that circHIPK3 can function as oncogenes, and may have an effect on tumor growth. In fact, plenty of investigations have indeed revealed the critical role of circHIPK3 in the growth, invasion, metastasis and resistance in the various cancers, such as lung cancer, bladder cancer, colorectal cancer, hepatocellular carcinoma, glioma, prostate cancer and osteosarcoma [15]. More importantly, Ba Y et al [16] shows that circHIPK3 enhances proliferation and metastasis of ESCC via modulation of miR-599/c-MYC axis, which indicates that circHIPK3 can also sponge miRNAs to regulate the progression of ESCC.

In our pre-experiment, we found that microRNA-124 (miR-124) was the most frequently verified target gene of circHIPK3 based on the Circ2Disease’s miRNA recognition element experiment results in circHIPK3 datasets (http://bioinformatics.zju.edu.cn/Circ2Disease/index.html). MiR-124 is reported to be down-regulated in both ESCC tissue and cells, which is strongly associated with the progression as well as prognosis of ESCC [17,18]. Furthermore, Zeng B et al [19] has been revealed that miR-124 can be sponged by circRNA_2646, which enhances the progression of ESCC. Based on these findings, we speculated that circHIPK3 may sponge miR-124 to modulate the progression of ESCC. Thus, in the present study, we aimed to explore whether circHIPK3 may sponge miR-124 to modulate the progression of ESCC, as well as its relevant mechanisms. We hope our results can lay an academic foundation for the early detection and treatments of ESCC, and even esophageal cancer.

## Materials and methods

### Patients and tissue specimens

The collection and manipulation of human patients’ tissues were approved by the Human Research Ethical Committee of Shenzhen Second People’s Hospital, The First Affiliated Hospital of Shenzhen University. Tissues used for research were collected from 32 patients with ESCC who underwent resection at Shenzhen Second People’s Hospital from 2018 to 2020. All the patients signed informed consent forms. The tissues were obtained and instantly transported in an ice box to the laboratory. Half part of the tissue was frozen in liquid nitrogen for RNA and protein extraction, and the other part was fixed in formaldehyde solution for RNAscope analysis.

### *Real time-quantitative PCR (RT-qPCR) analysis and* in situ *RNA detection*

Total RNA was extracted by Trizol (DP424, Tiangen, Beijing, China) from tissues and cells. The cDNA and RT-qPCR assays were performed using FastKing Real Time One Step RT-qPCR Kit (SYBR Green) (FP313, Tiangen) in the LightCyclerR480II System (Roche, Basel, Switzerland) following the manufacturer’s instructions. The conditions of PCR reaction were 40 cycles of pre-denaturation at 95°C for 1 min, denaturation at 95°C for 20s, annealing/extension at 60°C for 35s. The calculation of gene expression was conducted by using a 2^−ΔΔCt^ method [20]. β-actin or U6 was used as an internal control. The primers used in this study were as follows: circHIPK3-F: CCAGTGACAGTTGTGACAGCTACC, circHIPK3-R: GCCAAACGTGCCTCGACCAAG; β-actin-F: CTCCATCGTCCACCGCAAATGCTTCT, β-actin-R: GCTCCAACCGACTGCTGTCACCTTC; miR-124-F: 5′-GCTAAGGCACGCGGTG-3′, miR-124-R: 5′-GTGCAGGGTCCGAGGT-3′; U6-F: 5′-ATTGGAACGATACAGAGAAGATT-3′, U6-R: 5′-GGAAC -GCTTCACGAATTTG-3′. RNAscope manual procedure was conducted at Shenzhen Hospital laboratories following standard protocol (2018) as previously described with minor modifications [21].

### Nucleic acid electrophoresis and treatment with RNase R

The cDNA and gDNA PCR products were detected by 2% agarose gel electrophoresis. The DNA was separated by electrophoresis at 180 V for 15 min. The results were then illustrated by UV irradiation. RNase R treatment was performed as the previous description [22]. In brief, 2 mg of RNA was incubated with or without 5 U/mg RNase R (Sigma, St. Louis, MO, USA) for 15 min at 37°C, and then, the purified RNA was examined by RT-qPCR.

### Cell culture and shRNA transfection

KYSE-150 and KYSE-410 cells were gifts from Professor Xinchen Sun (Department of Radiology, the First Affiliated Hospital of Nanjing Medical University). The ECA-109, KYSE180 cells and a normal human esophageal epithelial cell line (HEEC) were purchased from GeneChem (Shanghai, China). All cell lines were cultured with DMEM (SH30285.03, Hyclone, Logan, UT, USA) with 10% fetal bovine serum (FBS, SH30084.04, Hyclone) and 1% streptomycin-penicillin (ST488, Beyotime, Beijing, China) at 37°C in a 5% CO_2_ incubator. KYSE-150 and ECA-109 cells were transfected with circHIPK3 knockdown lentivirus (sh-circHIPK3) and negative control vectors (both from GENECHEM, Shanghai, China) following the standard manufacturer’s instructions. The stable knockdown cell lines were selected with 4 μg/ml of puromycin treatment after 72 h of transfection. The efficiency of knockdown was tested by RT-qPCR. In addition, miR-124 mimic, si-AKT and negative controls (all from GENECHEM) were transfected into KYSE-150 and ECA-109 cells with Lipofectamine 3000 (Invitrogen, Carlsbad, CA, USA). The oligonucleotides of sh-circHIPK3 were as follows: CCGGGACGACCCTGACGGTACCGCGATATCTCAATTCGTTTGAGGTGGTTCAGTCGTCAATTTG. The sequences of miR-124 mimic were 5′-CCGUAAGUGGCGCACGGAAU-3′.

### Cell proliferation and colony formation assays

To conduct cell proliferation assays, Cell Counting Kit-8 kit (CCK-8, Dojindo, Kumamoto, Japan) and EdU Apollo® 567 *In Vitro* Imaging Kit (C10310-1, Ribobio, Guangzhou, China) were used according to the manufacturer’s instruction. In brief, cells were sowed into 96-well plates with 3 × 10^3^ cells per well and maintained at 37°C with 5% CO_2_ overnight. Then, each well of plates was supplemented with 10 μl of CCK-8 regents and further incubated at 37°C for 2 h. The optical density (OD) at 450 nm was detected by a microplate reader (Thermo Fisher Scientific, Waltham, MA, USA). To conduct the colony formation assay, 2.5 × 10^3^ cells were seeded into six-well plate. Ten days later, the colonies were fixed and stained with formaldehyde and crystal violet according to the manufacturer’s instruction (ST081, Beyotime). Then, visible colonies were photographed (Nikon, Tokyo, Japan) and calculated.

### Transwell analysis

The KYSE-150 and ECA-109 cells were prepared into serum-free cell suspension. Each Transwell chamber (Corning Company, New York, NY, USA) was inoculated with 2 × 10^4^ cells, and the lower Transwell chamber was added with 500 μl serum containing DMEM. The cells were incubated in 5% CO_2_ incubator at 37°C for 24 h, the cells were wiped with cotton swab, fixed with 4% paraformaldehyde for 30 min, and stained with 0.1% crystal violet for 20 min at room temperature. Five visual field cells were randomly selected under microscope, photographed and counted.

### Western blotting

Western Blotting was performed as described previously [23]. In brief, total proteins were obtained using a Total Protein Extraction Kit (BC3711, Solarbio, Beijing) and quantified with the BCA protein quantification kit (ab102536, Abcam, Cambridge, UK) according to the manufacturer’s specifications. Protein samples were dissolved and then electrically transferred to a PVDF membrane (EMD Millipore, Billerica, MA, USA). The antibodies contained E-cadherin (1:2000, 20,874-1-AP, Proteintech), Vimentin (1:2000, 10,366-1-AP, Proteintech), AKT3 (1:2000, 22,028-1-AP, Proteintech, Wuhan, China), GAPDH (1:100,000, 66,009-1-Ig, Proteintech) and Goat Anti-Rabbit (1:5000, SA00001-1, Proteintech). The protein expression was visualized by an ECL assay (P0018S, Beyotime).

### Renilla luciferase

The fragment of *AKT3* 3’ UTR containing the binding site of miR-124 was spliced to the 3’-end of the Renilla luciferase reporter gene. The wild-type or mutant circHIPK3 and miR-124 binding sites were sub-cloned into psiCHECK-2 system (Promega, Madison, USA), and subsequently transfected by Lipo3000 (Invitrogen). After transfection of 48 h, cells were collected to measure the luciferase activity by a microplate reader (Thermo Fisher Scientific, Waltham, MA, USA).

### Animals

All the procedures were carried out sternly based on the Guide for the Care and Use of Laboratory Animals [24] and the Guide for the Care and Use of Laboratory Animals and approved by the Ethics Committee of Shenzhen Second People’s Hospital, The First Affiliated Hospital of Shenzhen University [Number: SYXK (YUE) 2019–0122]. A total of 50 BALB/c nude mice were bought from Vital River (Beijing, China) and assigned to two groups, including NC group, in which mice were injected with ECA-109 cells) and sh-circHIPK3 group, in which mice were injected with ECA-109 cells transfected with circHIPK3 knockdown lentivirus. 200 µl of the above cell suspension containing 2 × 10^5^ cells was injected into the left or right back of each mouse. Tumor sizes and tumor volume were measured as described previously [25]. After four weeks, mice were euthanized with carbon dioxide for the collection of tissue samples.

### Statistical analysis

Statistical analyses were performed using Graphpad Prism software version 8.0 (USA). Experimental data are described as mean ± SEM. The significance of the observed differences was measured via the Student’s t-test or chi-square test. *P* < 0.05 was served to be statistically significant.

## Results

It has been demonstrated that circHIPK3 can sponge miRNAs in a variety of cancers, including ESCC, indicating that circHIPK3 can regulate the cancer progression through sponging miRNAs. In our pre-experiment, we found that miR-124 was the most frequently verified target gene of circHIPK3 based on the Circ2Disease’s miRNA recognition element experiment results in circHIPK3 datasets (http://bioinformatics.zju.edu.cn/Circ2Disease/index.html). Based on these findings, we speculated that circHIPK3 may sponge miR-124 to modulate the progression of ESCC. Thus, in the present study, we aimed to explore whether circHIPK3 may sponge miR-124 to modulate the progression of ESCC, as well as its relevant mechanisms. To achieve this goal, a luciferase reporter experiment was used to determine the binding sites between circHIPK3 and miR-124, as well as miR-124 and *AKT*. In addition, the role of circHIPK3 in the proliferation, migration and EMT of ESCC was explored by interference of circHIPK3 both in KYSE-150 and ECA-109 cell lines and xenografted nude mice.

### The expression pattern of circHIPK3 in ESCC tumor tissues

**Figure 1.**
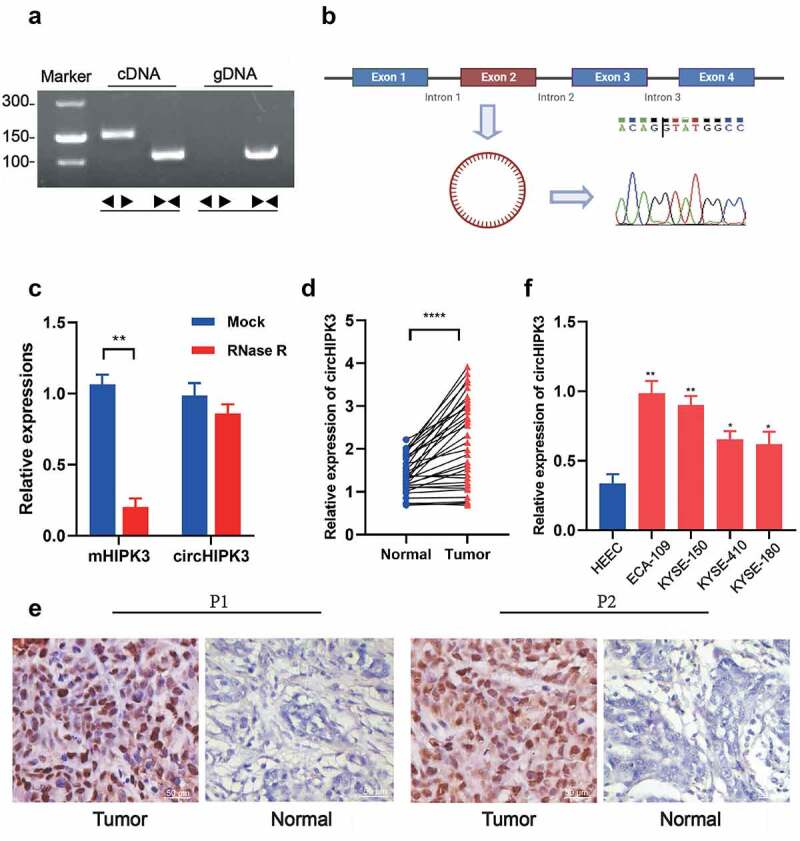
circHIPK3 validation and expression in ESCC tissues and cell lines. (a) The existence of circHIPK3 was validated in ECA-109 cell lines by RT-qPCR. Divergent primers amplified circHIPK3 in cDNA but not genomic DNA (gDNA). (b) Schematic illustration showing the circularization of HIPK3 exon 2 forming circHIPK3 (blue arrow). The presence of circHIPK3 was confirmed by Sanger sequencing. (c) The expression of circHIPK3 and HIPK3 mRNA in ECA-109 cells treated with or without RNase R was detected by RT-qPCR. (d) The expression of circHIPK3 was detected by real-time PCR in 32 pairs of ESCC and normal tissues. (e) Representative RNAscope images of circHIPK3 expression in ESCC tissues and adjacent normal tissue of two patients (P1 and P2). (f) The expression of circHIPK3 in ESCC cell lines was determined using RT-qPCR. (** *P* < 0.01, ****P < 0.0001, Student’s t-test.).

To understand the functional roles of circHIPK3 in ESCC, we first used RT-qPCR and RNAscope to examine the expression of circHIPK3 in tumor tissues and adjacent tissues. circHIPK3 was amplified in cDNA and gDNA (genomic DNA) from ESCC tissues using divergent primers that one included a divergent primer for the specifical enhancement of circHIPK3 and the other comprised an antisense primer for HIPK3 mRNA detection. The results showed that circHIPK3 was amplified in cDNA and no amplification product was found in gDNA (Figure 1(a)). Sanger sequencing confirmed the cyclization site sequence (Figure 1(b)). Moreover, RNase R treatment significantly reduced the expression of linear HIPK3 mRNA, but not changed the expression level of circHIPK3 mRNA, confirming the stability of circHIPK3 (Figure 1(c)). The expression of circHIPK3 was then shown to be significantly higher in ESCC tissues than that in surrounding normal tissues (Figure 1(d)). Additionally, RNAscope analysis demonstrated the increased expression of circHIPK3 in two ESCC patients’ cancerous tissues compared with that in the surrounding normal tissues (Figure 1(e)). More importantly, patients with enhanced circHIPK3 expression had lymph node metastases, a greater tumor size, and a poor differentiation of the tumor (Table 1). circHIPK3 expression was expectedly upregulated in ESCC four cell lines, especially in KYSE-150 and ECA-109 cell lines compared to the normal human esophageal epithelial cell line (HEEC) (Figure 1(f)). Thus, KYSE-150 and ECA-109 cell lines were employed for subsequent investigations. In summary, these data revealed that stable circHIPK3 was highly expressed in both ESCC tissues and cells.

**Table 1.** Relationship between circHIPK3 expression in patients with ESCC and clinicopathologic characteristics

		circHIPK3 expression		
Feature	No.	High	Low	P value
Age(years)				
≥60	23	15	8	0.612
<60	9	5	4	
Tumor size(cm)				
≥4.0	20	16	4	0.008*
<4.0	12	4	8	
Tumor differentiation				
Poorly	19	15	4	0.020*
High	13	5	8	
Lymph node metastasis				
N0-N1	14	6	8	0.043*
N2-N3	18	14	4	
TNM Stage				
I/II	10	5	5	0.325
III/IV	22	15	7

### *CircHIPK3 promotes proliferation and migration of ESCC both* in vitro *and* in vivo

**Figure 2.**
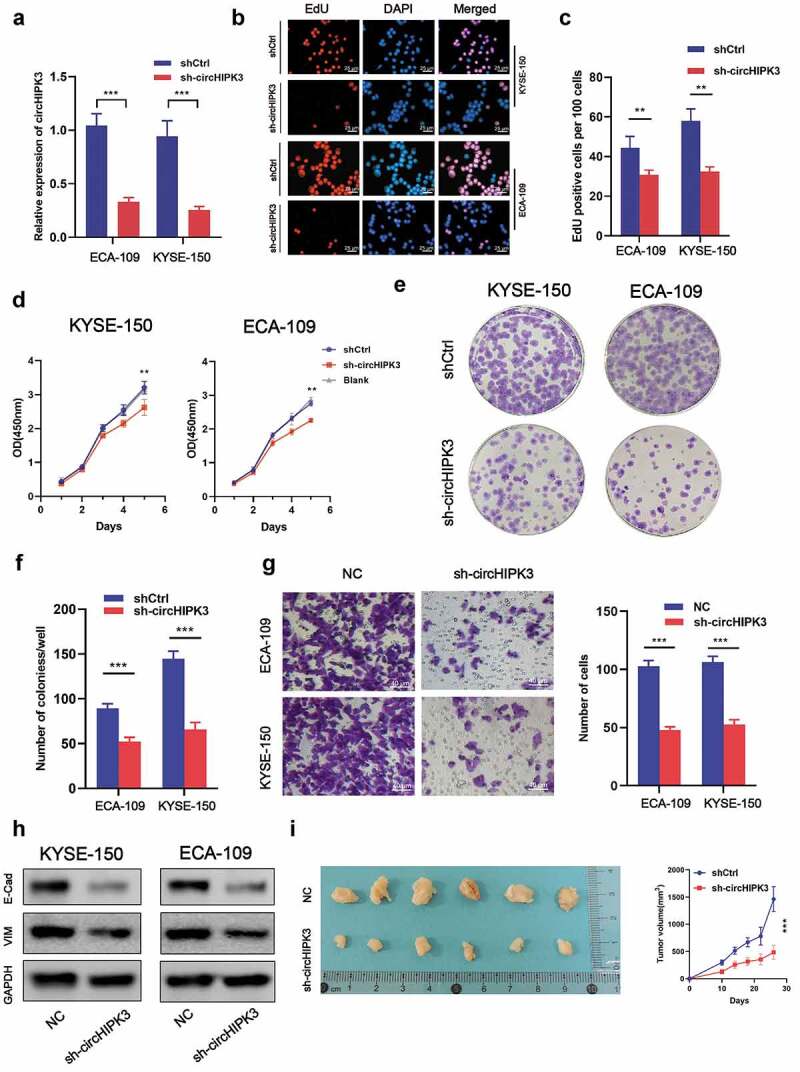
circHIPK3 Promotes ESCC Cells Tumorigenicity *in vitro* and *in vivo*. (a) Expression level of circHIPK3 knockdown efficiency in ECA-109 and KYSE-150 cell lines was detected by RT-qPCR. (b-d) The influences of circHIPK3 knockdown on cell proliferation were confirmed using the CCK-8 assay and EdU assay. (e-f) The representative picture of colony formation assay, and the quantification of colonies per well. (g) Transwell analysis was used to determine the effects of circHIPK3 on migration in ECA-109 and KYSE-150 cell lines. (h) WB exhibited the protein levels of E-cadherin and Vimentin in ESCC cells transfected with shCtrl or sh-circHIPK3. (i) Knockdown of circHIPK3 effectively inhibited ECA-109 cells subcutaneous tumor growth and migration in nude mice. (** *P* < 0.01, *** *P* < 0.001, Student’s t-test.).

To determine if circHIPK3 expression was associated with ESCC progression, shRNAs targeting circHIPK3 were transfected into KYSE-150 and ECA-109 cells, respectively, by lentivirus infection. RT-qPCR analysis revealed that shRNAs significantly decreased the expression of circHIPK3 (Figure 2(a)), which indicated the practicable interference efficiency of circHIPK3 shRNAs. EdU and CCK-8 results revealed that circHIPK3 knockdown inhibited cell growth in both the KYSE-150 and ECA-109 cell lines (Figure 2(b-d)). Additionally, downregulation of circHIPK3 expression lowered growth potential in both cell lines, as less colonies formed after 9 days compared to the shCtrl group (Figure 2(e,f)). Meanwhile, silencing circHIPK3 expression dramatically decreased the migration of KYSE-150 and ECA-109 cells (Figure 2(g)). Interference of circHIPK3 expression significantly decreased the expression of E-cadherin and vimentin (Figure 2(h)), which suggested that circHIPK3 shRNAs inhibited the epithelial-mesenchymal transition (EMT). Furthermore, *in vivo* assays also confirmed that silencing circHIPK3 expression suppressed the tumor growth (Figure 2(i)). Based on these results, we concluded that downregulation of circHIPK3 restrained proliferation and migration of ESCC both *in vitro* and *in vivo*.

### CircHIPK3 interacts with miR-124 to meditate ESCC cell proliferation and migration

**Figure 3.**
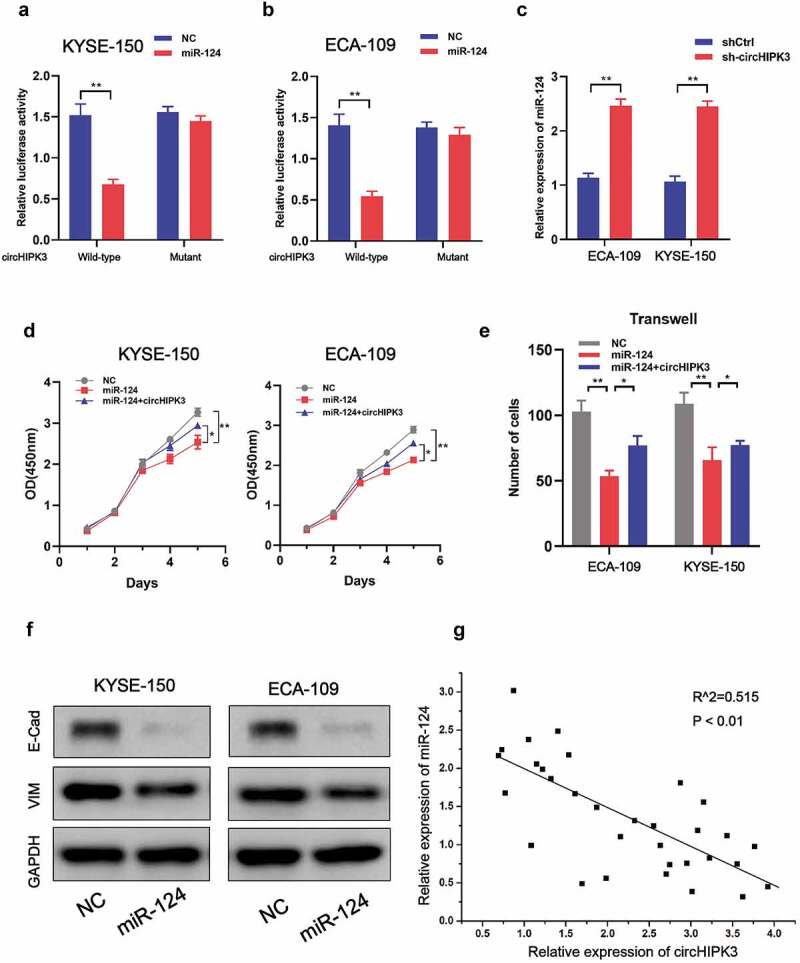
circHIPK3 interacts with miR-124 to meditate ESCC cells proliferation and migration. (a-b) Luciferase report analysis of ESCC cells co-transfected with miR-124 or control and circHIPK3 WT or circHIPK3 MUT. (c) RT-qPCR was used to measure miR-124 expression in ESCC cells with shCtrl or circHIPK3 knockdown. (d) CCK-8 analysis of ESCC cell proliferation after co-transfection with NC-mimics, miR-124, or circHIPK3 as indicated. (e) Transwell analysis of ESCC cell migration after co-transfection with NC-mimics, miR-124, or circHIPK3 as indicated. (f) WB exhibited the protein levels of E-cadherin and Vimentin in ESCC cells transfected with NC-mimics or miR-124. (g) A negative correlation between circHIPK3 and miR-124 in ESCC tissues. (** *P* < 0.01, Student’s t-test.).

Since circHIPK3 has been shown to act as a miRNA sponge in a variety of malignancies, we selected candidate miRNAs using Circ2Disease’s miRNA recognition element experiment results in circHIPK3 datasets (http://bioinformatics.zju.edu.cn/Circ2Disease/index.html). The Circ2Disease database indicated that miR-124 was the most frequently verified target gene of circHIPK3. Luciferase reporter assays in KYSE-150 and ECA-109 cells illustrated that co-transfection of miR-124 and circHIPK3 significantly decreased luciferase activity compared to co-transfection of miR-124 and circHIPK3-MUT (Figure 3(a,b)), which indicated the direct link between miR-124 and circHIPK3. In addition, miR-124 expression was increased upon circHIPK3 silencing (Figure 3(c)). Moreover, overexpression of miR-124 significantly inhibited cell proliferation and migration of KYSE-150 and ECA-109 cells, which could be rescued by co-transfection with circHIPK3 (Figure 3(d,e)). Also, upregulation of miR-124 decreased E-cadherin and vimentin expression (Figure 3(f)). Furthermore, a negative connection between miR-124 and circHIPK3 was identified in real-world tumor tissues (Figure 3(g)). Therefore, these outcomes suggested that CircHIPK3 could directly interact with miR-124 to regulate ESCC cell growth and migration.

### AKT3 *is a target of miR-124 and is meditated by circHIPK3*

**Figure 4.**
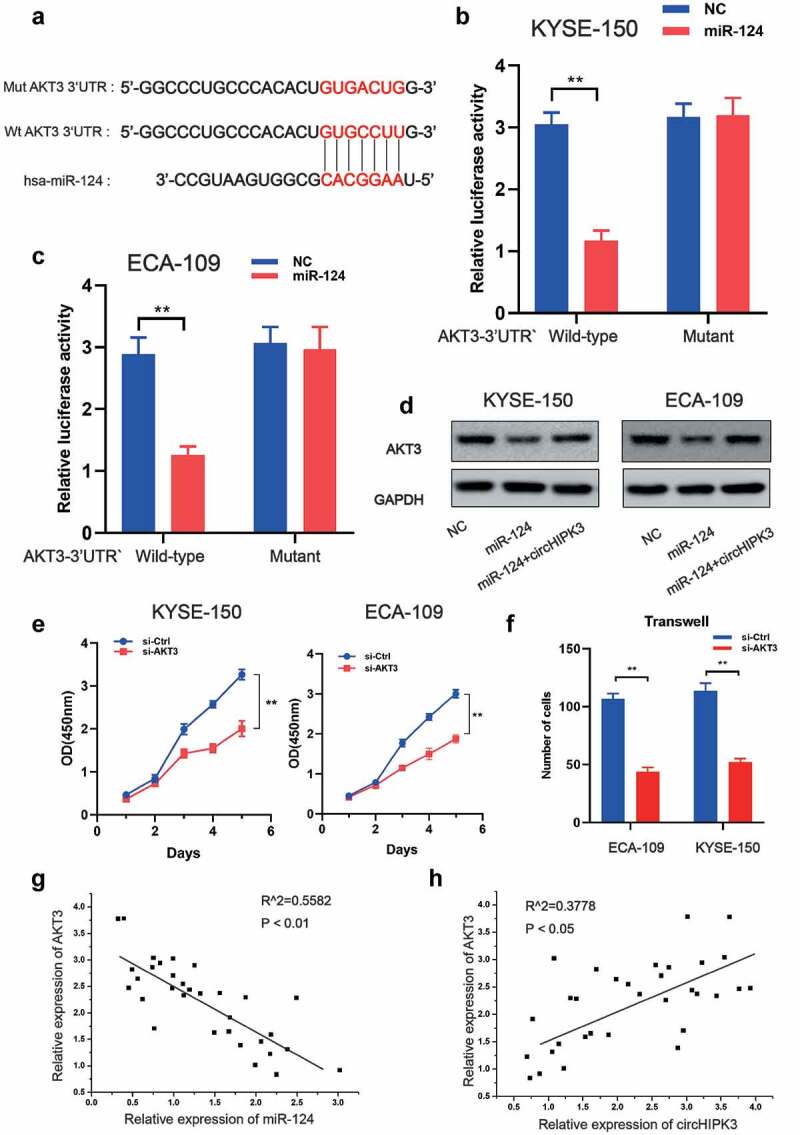
AKT3 is the target of miR-124 in ESCC cells. (a) Putative and mutant binding sites of miR-124 on AKT3. (b-c) Luciferase report analysis of ESCC cells co-transfected with miR-124 or control and AKT-3UTR-WT or AKT-3UTR-MUT. (d) WB was used to measure AKT3 expression in ESCC cells with miR-124 or circHIPK3. (e) CCK-8 analysis of ESCC cell proliferation after co-transfection with si-Control or si-AKT3 as indicated. (f) Transwell analysis of ESCC cell migration after co-transfection with si-Control or si-AKT3 as indicated. (g-h) A correlation between circHIPK3, miR-124 and AKT3 in ESCC tissues. (** *P* < 0.01, Student’s t-test.).

Notably, the RTK-MAPK-PI3K signaling pathway is frequently dysregulated in ESCC by a variety of molecular pathways, implying that the genes implicated in this signaling pathway may be miR-124 target genes. *AKT3* was identified as a prime target of miR-124 based on bioinformatics prediction using TargetScan (http://www.targetscan.org/vert 72/), as it contained a highly conserved complementary miR-124-binding site in its 3’ UTR across vertebrates from Lizard to Human (Figure 4(a)). We used luciferase reporter assays in KYSE-150 and ECA-109 cells to confirm miR-124 function at the *AKT3* 3’ UTR. As illustrated in Figure 4(b,c), co-transfection of miR-124 with wild-type *AKT3* 3’ UTR vectors significantly decreased luciferase activity. Furthermore, miR-124 overexpression suppressed *AKT3* expression, whereas circHIPK3 overexpression reversed this effect (Figure 4(d)). Clearly, silencing *AKT3* expression inhibited cell growth and migration (Figure 4(e,f)). To further substantiate the synergy between circHIPK3/miR-124/*AKT3* in ESCC, we examined the association between circHIPK3/miR-124/*AKT3* expression in ESCC tumor tissues. It is worth noting that *AKT3* expression was positively connected with circHIPK3 expression and negatively associated with miR-124 expression respectively (Figure 4(g,h)), implying that circHIPK3 may regulate *AKT3* expression by sponging miR-124. Thus, these data indicate that circHIPK3 regulated the progress of ESCC via miR-124/*AKT3* axis.

## Discussion

ESCC is one of the most lethal malignant tumors in the world, and its treatment is limited [3,26]. Additionally, the basic mechanism behind the onset and progression of ESCC remains largely unclear. As a result, it is critical to investigate the biological mechanisms underlying ESCC in order to find molecular biomarkers for early detection and prognosis. CircRNAs are a type of noncoding RNA (ncRNA) with a closed-loop structure and no coding region [27]. Emerging evidence has revealed that circRNAs play a critical role in different types of human cancers [28]. CircRNAs act as a miRNA sponge, influencing numerous biological processes by changing RNA splicing, chromatin structure, and mRNA stability [29,30]. In the present study, the expression of circHIPK3 was upregulated in both ESCC tissues and cells, which was associated with a poor prognosis. Mechanically, circHIPK3 inhibited growth, migration and EMT of ESCC via miR-124/*AKT3* axis.

Extensive reports have been demonstrated that the expression of circHIPK3 is upregulated in a variety of cancer, such as prostate cancer [31], breast cancer [32], ovarian cancer [33], colorectal cancer [34], lung cancer [35] and gastric cancer [36]. It has been also revealed that high level of circHIPK3 is strongly associated with unfavorable clinicopathological features in patients with CRC [34]. Besides, upregulation of circHIPK3 level indicating the poor prognosis has been confirmed in gastric cancer [37] and epithelial ovarian cancer [38]. In the present study, the expression of circHIPK3 was also upregulated in both ESCC tissues and cells, which was tightly involved in the lymph node metastases, tumor size, and a poor differentiation of the tumor, in line with the previous study [16]. Moreover, overexpression of circHIPK3 generally contributes to the development of tumors. For instance, upregulation of circHIPK3 enhances the proliferation, migration and invasion of prostate cancer cells [31] and breast cancer cells [32]. Overexpression of circHIPK3 promotes the ovarian cancer cell proliferation and inhibits its apoptosis [33]. Similar to these findings, our results also showed that knockdown of circHIPK3 suppressed esophageal cancer cell proliferation, migration and epithelial-mesenchymal transition (EMT) *in vitro*, as well as the growth *in vivo*. Taken together, the expression of circHIPK3 was upregulated in ESCC, which indicated a poor prognosis and promoted the progression of ESCC.

It has been revealed that circHIPK3 can act as a sponge to competitively bind with miRNAs that affects the progress of cancers. CircHIPK3 sponges miR-326 to enhance the progression of breast cancer [32]. CircHIPK3 regulates prostate cancer progression through targeting miR-448/MTDH axis [31]. CircHIPK3 modulates miR-1207-5p/FMNL2 signal that promotes colorectal cancer cells proliferation and metastasis [34]. Moreover, circHIPK3 can regulate ESCC cell proliferation via the miR-599/c-MYC axis [16]. In the current study, the binding sites between circHIPK3 and miR-124 were predicated by the bioinformatics database and verified by luciferase reporter assays. MiR-124 plays a pivotal role in ESCC. It has been reported that a genetic variant in miR-124 reduces the susceptibility to ESCC in a Chinese Kazakh population [39]. Downregulation of miR-124 level in ESCC is also reported in previous studies, which indicates a poor prognosis [17,40]. Furthermore, miR-124 can be sponged by circRNA_2646 to enhance the ESCC progression [19]. Here, a negative connection between miR-124 and circHIPK3 was identified in real-world tumor tissues, thus, co-transfection with circHIPK3 significantly antagonized the miR-124 mimic-induced the inhibition of proliferation and migration of KYSE-150 and ECA-109 cells in the present study. Thus, these outcomes expounded that circHIPK3 interacted with miR-124 to meditate ESCC cell proliferation and migration.

Additionally, our research established for the first time that *AKT3* is a miR-124 target gene, based on the consensus that miRNAs could directly bind to the down-stream target mRNA and further depresses the expression of the mRNA. *AKT3* encodes an AGC kinase family serine/threonine protein kinase [41] that participates in signaling pathways involved in cell proliferation, oncogenesis, survival, migration, and intracellular protein trafficking [42,43]. Mutations in *AKT3* have been associated with tumor development and migration. Gain of function mutations in *AKT3* were discovered in a variety of cancer types, including breast cancer and endometrial cancer, where the PI3K signaling pathway was implicated [44,45]. Moreover, Shi N et al [46] reports that circ-PRKCI functions as a competitive endogenous RNA to modulate AKT3 expression via sponging miR-3680-3p in ESCC. In the present study, *AKT3* was confirmed to be directly targeted by miR-124 and negatively associated with the miR-124 expression. Hence, overexpression of miR-124 notably reduced the relative protein levels of AKT3, which was obviously reversed by co-transfection of circHIPK3. Moreover, the expression of *AKT3* was positively related in the circHIPK3 levels. Furthermore, downregulation of *AKT3* expression dramatically inhibited ESCC cell proliferation and migration, which were consistent with those seen in other cancers [44]. Therefore, these data indicate that circHIPK3 regulated the progress of ESCC via miR-124/*AKT3* axis.

Nevertheless, there are also several limitations in the current study. (1) The off-target effects are one of important focuses. Thus, except for the negative control, the off-target controls including simultaneous experiments with siRNAs of at least two targets, or rescue experiments need to be conducted in our subsequent studies. (2) Although the experiment was repeated three times in the current study, more replicates can be conducted in our subsequent studies. (3) Experiments performed in two cell line only fail to control for cell-dependent effects, hence, additional cells lines, such as TE 13 need to be examined to generalize the proposed mechanism in further studies.

## Conclusion

In summary, for the first time in the present study, our results indicated that circHIPK3 functioned as an oncogenic circRNA that enhanced ESCC tumorigenesis and progression through the miR-124/ *AKT3* pathway. The findings here indicate that a circHIPK3/miR-124/ *AKT3* axis may be a potential therapeutic target for ESCC.

## Supplementary Material

Supplemental Material

## Data Availability

The datasets used and/or analyzed during the current study are available from the corresponding author on reasonable request.
