# Supplementary material for: circHIPK3 regulates cell proliferation and migration by sponging microRNA-124 and regulating serine/threonine kinase 3 expression in esophageal squamous cell carcinoma
Source: Bioengineered. 2022 Apr 21;13(4):9767–80. doi: 10.1080/21655979.2022.2060776 (PMC9161938; doi:10.1080/21655979.2022.2060776)
Supplement: Supplemental Material [file KBIE_A_2060776_SM2113.zip › Supplementary document 1 Approval Of Ethic Committee.pdf]

Approval Of Ethic Committee

Date of Application 申请日期： 2019 年 9 月 25 日

The Project<br>项目名称	CircHIPK3 acts as an oncogene by sponging miR-124 and regulating AKT3 expression in esophageal squamous cell carcinoma.
Major Investigators<br>主要研究者	Da Yao, Shengcheng Lin
Department<br>专业&科室	Department of Thoracic Surgery
Approval NO.<br>伦理编号	SYXK（粤）2019-0122
Classification<br>研究分类	1 病理标本实验 Pathology specimens research (√)<br>2 人体实验 Human research ( )<br>3 动物组织或细胞实验 Animal tissue or cell experiments (√)<br>4 其他研究 Other research ( )
Conclusion<br>审查意见	【√ 】同意 Approved<br>【 】修正后同意 Agree after revision (Specify modification below or in accompany letter)<br>【 】不同意 Disagree (Specify reasons below or in accompany letter) ____________
Statement<br>审查声明	兹证明本研究的设计和方法符合相关法规和伦理原则的要求。伦理委员会批准本研究项目在本院执行。<br>This is to certify that the design and methods of the research are in accordance with the requirements of related regulations and procedures as well as the ethical principles. The IRB has approved the research to be conducted in our hospital.<br>深圳市第二人民医院<br>Shenzhen Second People's Hospital,<br>2019 年 9 月 25 日

深圳市第二人民医院 科研伦理委员会
