# Supplementary material for: circHIPK3 regulates cell proliferation and migration by sponging microRNA-124 and regulating serine/threonine kinase 3 expression in esophageal squamous cell carcinoma
Source: Bioengineered. 2022 Apr 21;13(4):9767–80. doi: 10.1080/21655979.2022.2060776 (PMC9161938; doi:10.1080/21655979.2022.2060776)

FIGG2 ECAD 135kDa

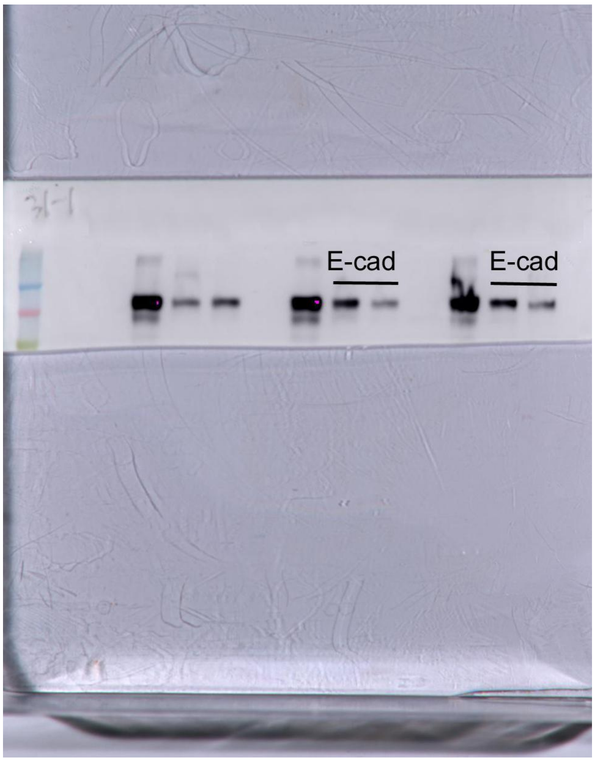

FIG2 Vimentin -57kDa

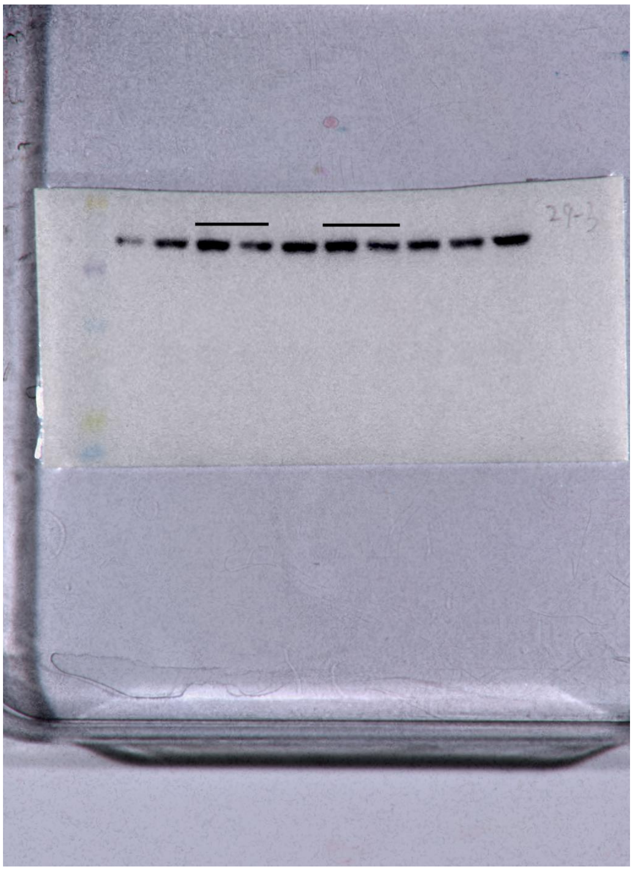

FIG2 GAPDH

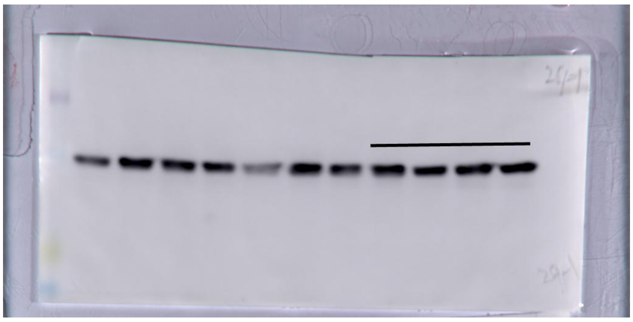

FIG3 E-CAD 135kDa

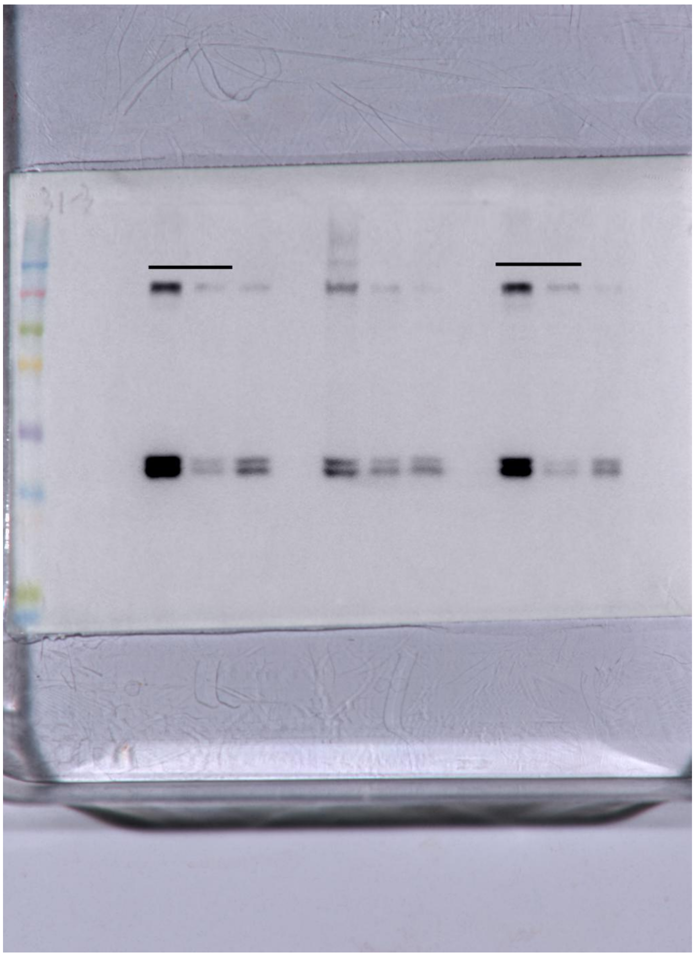

FIG3 Vimentin -57kDa

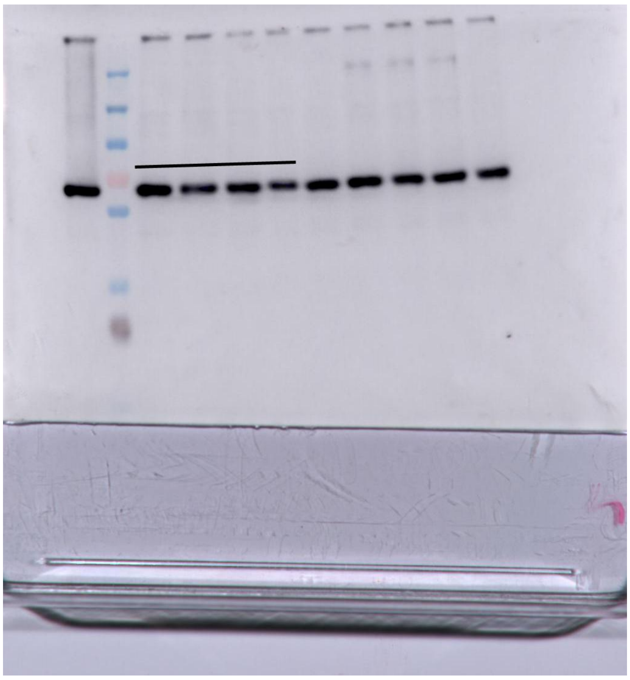

FIG3 GAPDH 36kDa

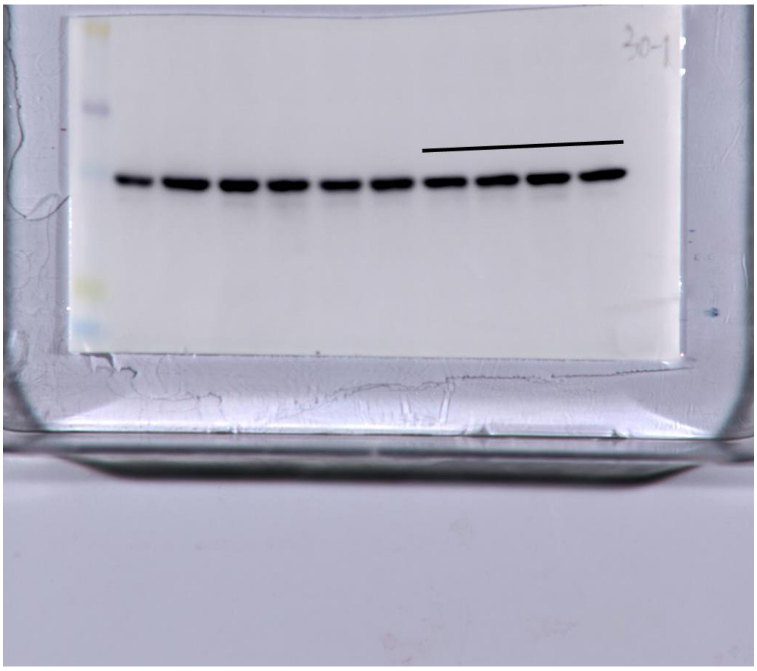

FIG4 AKT 60kDa

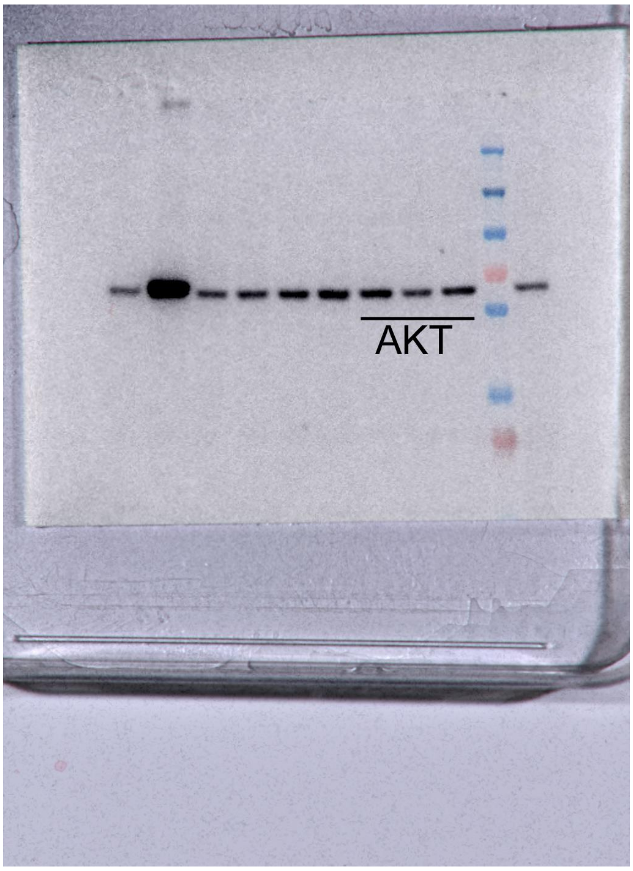

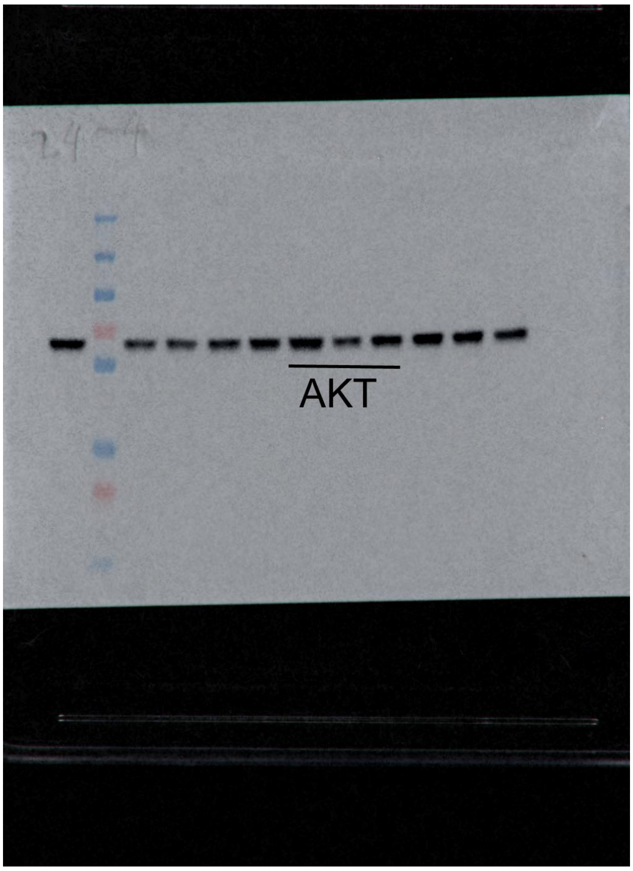

Fig4 GAPDH-36 kDa

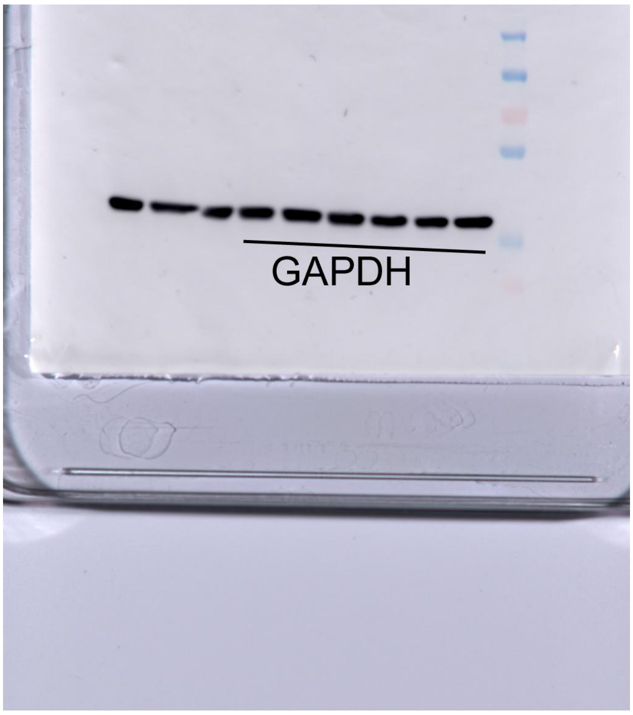

Supplement: Supplemental Material [file KBIE_A_2060776_SM2113.zip › Supplementary document 2 Original WB bands.pdf]
